# A NONTRADITIONAL PATH TOWARD A RESEARCH CAREER

**DOI:** 10.1093/geroni/igae098.1525

**Published:** 2024-12-31

**Authors:** Ketlyne Sol

**Affiliations:** University of Michigan, Ann Arbor, Michigan, United States

## Abstract

I graduated with my PhD in Clinical Psychology, where I received both clinical and research training. I specialized in rehabilitation psychology and received additional training in neuropsychology. I focused the bulk of my graduate training on developing strong clinical skills because I wanted the research I produced to be informed by clinical experience. My research training gave me a strong foundation in research methodology and statistical analysis. But I underestimated the business side of research and the culture of academia overall. When I decided to pursue dedicated research training in disparities in cognitive aging, it had to be a full-time commitment, as it had been with my clinical training. Focused research training helped me progress much better and faster than I would have been able to trying to juggle both a clinical and research career. I became much better at grant writing and at communicating my research. I also believe that no person is an island. So, I also credit my success to organically forming the right combination of formal and informal mentors, peers, and other friendships during each step of my career, along with maintaining relevant pre-existing kin- and friend-ships. I was told early on that a career in clinical research is a marathon, not a sprint, but it took me a bit of time to fully understand that statement. I have finally embraced the marathoner’s mindset because I enjoy this work and I intend to produce good research for a very long time.

